# Holographic Three-Dimensional Imaging of Terra-Cotta Warrior Model Using Fractional Fourier Transform

**DOI:** 10.3390/jimaging5080067

**Published:** 2019-07-26

**Authors:** Zhi-Fang Gao, Hua-Dong Zheng, Ying-Jie Yu

**Affiliations:** Department of Precision Mechanical Engineering, Shanghai University, Shanghai 200072, China

**Keywords:** holographic imaging, fractional Fourier transform, spatial light modulator

## Abstract

Holographic three-dimensional (3D) imaging of Terra-Cotta Warrior model using Fractional Fourier Transform is introduced in this paper. Phase holograms of Terra-Cotta Warrior model are calculated from 60 horizontal viewing-angles by the use of fractional Fourier transform (FRT). Multiple phase holograms are calculated for each angle by adding proper pseudorandom phase to reduce the speckle noise of a reconstructed image. Experimental system based on high-resolution phase-only spatial light modulator (SLM) is built for 3D image reconstruction from the calculated phase holograms. The texture of the Terra-Cotta Warrior model is rough. The calculation of rough texture is optimized in order to show better model details. The effects of computing distance and layer thickness on imaging quality are analyzed finally.

## 1. Introduction

Holographic display is a three-dimensional (3D) display method for showing 3D information of real objects. Holography can record the light wave information of objects in space and then perform optical redisplay [1]. Holographic 3D display can record the depth information of objects, so that it can improve the reality sense of 3D image. Valuable cultural relics are recorded and redisplayed by the holographic method in the protection of cultural relics. It can not only redisplay the material information of cultural relics, but also protect the original cultural relics from repeated exposure to the air. Holographic imaging can show three-dimensional information of cultural relics, which can show us more useful information, when compared with the traditional two-dimensional (2D) plane photo imaging method. Holograms for holographic display can be generated by optical holographic system or computational methods [2,3,4]. Computer generated hologram (CGH) has an important advantage [5]. Computing virtual object hologram avoids the complicated and strict operation of optical hologram acquisition. Recently, some methods for generating CGH from three-dimensional objects have been proposed. [6,7,8,9,10]. There are three types: pure amplitude hologram, pure phase hologram, and complex hologram (including amplitude and phase information), according to the wavefront information in the hologram plane. Kinoform is a phase hologram with high diffraction efficiency [11]. It can reduce the noise of the reconstructed image, but the reconstruction error of the amplitude distribution in the image plane is introduced, because the amplitude of the wavefront in the hologram plane is neglected. The distribution of this kind of noise is usually irregular. We also call it speckle noise or random noise. This kind of noise will destroy the texture details of the image, reduce the quality of the image, and reduce the comfort degree of human observation. [12]. The algorithm of reducing speckle noise arises in order to improve the image quality. These methods mainly include two types: one is for the processing of reconstructed images and the other is focused on heterogeneous optimization to improve the quality of reconstructed images. In the first processing method, random diffusers (such as ground glass plates) are usually used to reduce the speckle noise in holographic reconstruction settings [13,14]. Moving aperture can reduce that speckle noise that is caused by time averaging effect [15]. In [16], Zhang et al. proposed the method of displaying full color 3D objects using fractional Fourier transform (FRT). They redisplayed a toy model and analyze the noise by image superposing method. However, the effect of computing distance on image quality was not analyzed, and interval of slices may affect image quality of objects with rough surface. We hope that we can redisplay more details when we redisplay the Terra-Cotta Warrior model, and analyze the effect of computing distance on image quality.

This paper presents a method to realize holographic 3D imaging of Terra-Cotta Warrior model using FRT. By superimposing the image reconstructed by multiple phase holograms, the noise in the holographic reconstruction image can be significantly reduced. Section 2 will describe methodological theory. Section 3 is the digital simulation and photoelectric reconstruction experiments, and the results are presented and analyzed. Additionally, in Section 3, the Terra-Cotta Warrior model surface texture is more complex and contains more information, in order to better observe the model when reproducing, we studied the calculation optimization of the Terra-Cotta Warrior model texture. The discussion and conclusion will be given finally.

## 2. Principle of The Proposed Method

### 2.1. Spatial Coordinate Transformation for Calculating Holograms of Terra-Cotta Warrior Model

**Figure 1:**
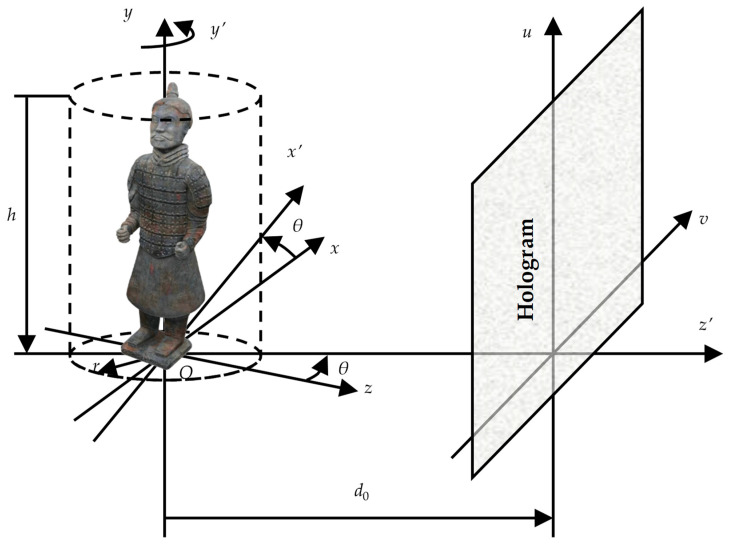
Coordinates for one-dimensional rotation transformation.

Generally, holograms for 360-degree viewing angle of the model are needed if we want to see 360-degree information of Terra-Cotta Warrior model. A cylindrical coordinate system is established to calculate 360-degree holograms of the Terra-Cotta Warrior model. As shown in Figure 1, the Terra-Cotta Warrior model is in the center of the cylinder. The radius and height of the cylinder are set to *r* = 30 mm and *h* = 200 mm, respectively. The resolution is set as 195.3 μm in the horizontal, as well as vertical, direction of object plane. The hologram plane is set at the distance of *d*_0_ in the coordinate system $\left(x^{\prime }y^{\prime }z^{\prime }\right)$. We calculate the holograms of the model along a circle route line, with 60 viewpoints at intervals of 6 degrees [17].

(1) $$\left(\begin{array}{c} x^{\prime }\\ z^{\prime } \end{array}\right)=\left(\begin{array}{cc} cos\theta & -sin\theta \\ sin\theta & cos\theta \end{array}\right)\left(\begin{array}{c} x\\ z \end{array}\right)$$

(2) $$y^{\prime }=y$$

where $\left(x^{\prime }Oy^{\prime }\right)$ plane is in parallel with (u,v) hologram plane, *θ* is the rotation angle along the $y\left(y^{\prime }\right)$ axis, and the distance between $y\left(y^{\prime }\right)$ axis and the hologram plane is *d*_0_.

### 2.2. FRT and Hologram Generation

The optical FRT of a two-dimensional object function can be expressed as [16]:

(3) $$E\left(u,v\right)=F^{p}\left\{f\left(x,y\right)\right\}=\iint f\left(x,y\right)\times \exp\left[j\pi \frac{x^{2}+y^{2}+u^{2}+v^{2}}{\lambda f_{e}\tan\left(p\pi /2\right)}-j2\pi \frac{xu+yv}{\lambda f_{e}\sin\left(p\pi /2\right)}\right]dxdy,$$

where $F^{p}\left\{\cdot \right\}$ denotes the FRT with an order p (0<|p|<2). f(x,y) and E(u,v) are the complex amplitude distributions in the object plane and fractional Fourier plane. *λ* is the wavelength of illuminating light. $f_{e}$ is the standard focal length. 

Two Formulas (4) and (5) are added in order to more clearly explain. These two formulas can be used to better explain the fractional Fourier calculation principle.

(4) $$Q=\sin\left(\frac{p\pi }{2}\right),R=\tan\left(\frac{p\pi }{4}\right)$$

(5) $$f=\frac{f_{e}}{Q},d=Rf_{e}$$

**Figure 2:**
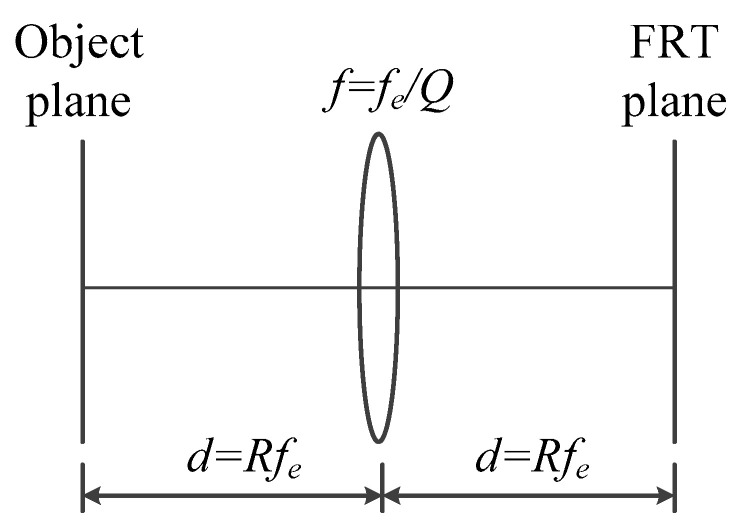
Lohmann type-I optical setup for performing a FRT.

Figure 2 is an optical principle model of Lohmann type-I FRT, when light emitted or reflected from an object propagates freely a distance $d=Rf_{e}$ from space. Light travels through a lens with a focal length of $f=\frac{f_{e}}{Q}$, and then propagates in another space at the same distance, which is symmetrical with the space through which the light first passes. When the object light reaches the focal distance of the lens, its Fourier transform can be obtained. The Fourier hologram can record not only the information of the object, but also the information of the system parameters, because of the characteristics of the Fourier transform.

**Figure 3:**
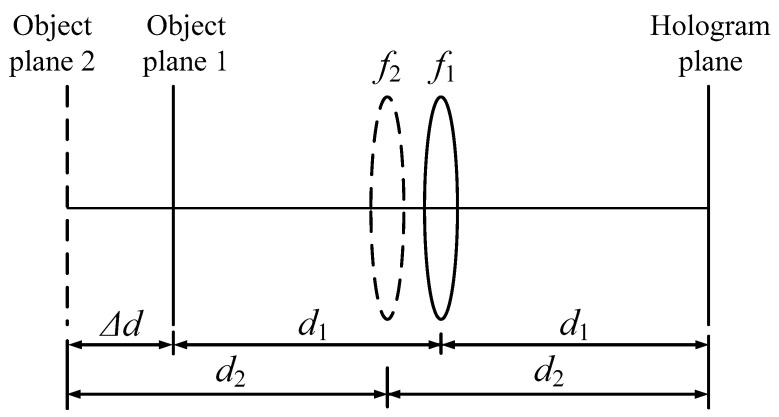
Optical configuration for generating FRT holograms of 3D objects.

According to the optical model of Lohmann type-I, we have proposed an improved optical model to analyze a three-dimensional object [16]. We assume that the depth of a three-dimensional object consists of many layers in different depths, as shown in Figure 3.

Suppose that we divide the object into *L* layers, that is, *L* layers of object planes. The distance from each layer to the lens is different. In Figure 3, we assume that the distance from the first layer to the lens is *d*_1_, and the distance from the second layer to the lens is *d*_2_. The distance between the two planes can be expressed as

(6) $$\Delta d=2\left(d_{2}-d_{1}\right)$$

According to Formulas (4) and (5), when the plane extends to the first plane, the distance from the plane to the lens is *d_i_*, and the standard focal length $f_{e}$ are given, the fractional order *p_i_* of the *i*th object plane can be expressed as

(7) $$p_{i}=4\tan^{-1}\left(d_{i}/f_{e}\right)/\pi$$

Due to the scaling factor $\lambda f_{e}=1$, for a give wavelength of 632.8 nm, $f_{e}$ = 1580 mm. Subsequently, the FRT of the *i*th object plane (0 < *i* < *L*) for a certain viewpoint of *θ* can be described as

(8) $$E_{\theta ,t,i}(u,v)={ℱ}^{p_{i}}\left\{f_{\theta ,i}(x,y)\right\}=\iint f_{\theta ,i}(x,y)\exp[j\varphi_{t}(x,y)]\times \exp[j\pi \frac{x^{2}+y^{2}+u^{2}+v^{2}}{\lambda f_{e}\tan(p_{i}\pi /2)}-j2\pi \frac{xu+yv}{\lambda f_{e}\sin(p_{i}\pi /2)}]dxdy,$$

where, $f_{\theta ,i}\left(x,y\right)$ is the complex amplitude of the *i*th object plane in the viewpoint of *θ* and $E_{\theta ,t,i}\left(u,v\right)$ is the contributed complex amplitude of the *i*th object plane for the *t*th hologram in the viewpoint of *θ*. *t* = 1, 2, 3…*T* (*T* is the total calculation times of holograms). $\varphi_{t}\left(x,y\right)$ is the dynamic-changed random phase factor for the *t*th hologram calculation process, which is used to smoothen the spatial spectrum distribution of the object information. By adding this random phase factor [0, 2π] to object planes, the contribution of all the surface layers to the hologram in the viewpoint of *θ* can be expressed by the following formula.

(9) $$E_{\theta ,t}\left(u,v\right)=\overset{L}{\underset{i=1}{\sum }}E_{\theta ,t,i}\left(u,v\right)$$

Thus, the *t*th FRT phase-only hologram of a 3D object is expressed as

(10) $$H_{\theta ,t}\left(u,v\right)=\arg\{E_{\theta ,t}\left(u,v\right)\}$$

where, arg{·} denotes the argument of the complex amplitude.

## 3. Digital Reconstruction and Electro-Optical Reconstruction Experiment

### 3.1. Digital Reconstruction

**Figure 4:**
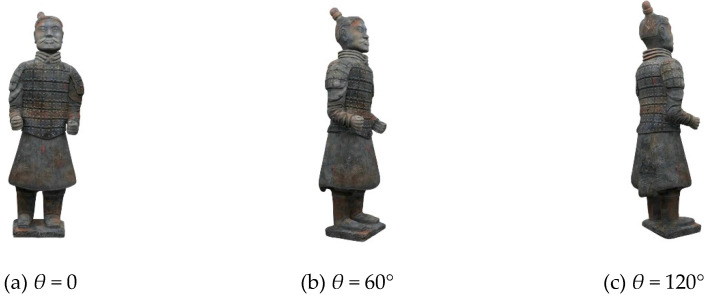

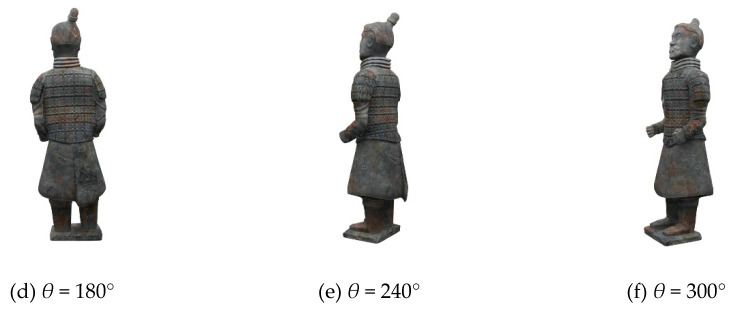
Original 3D images viewed at different angles.

**Figure 5:**
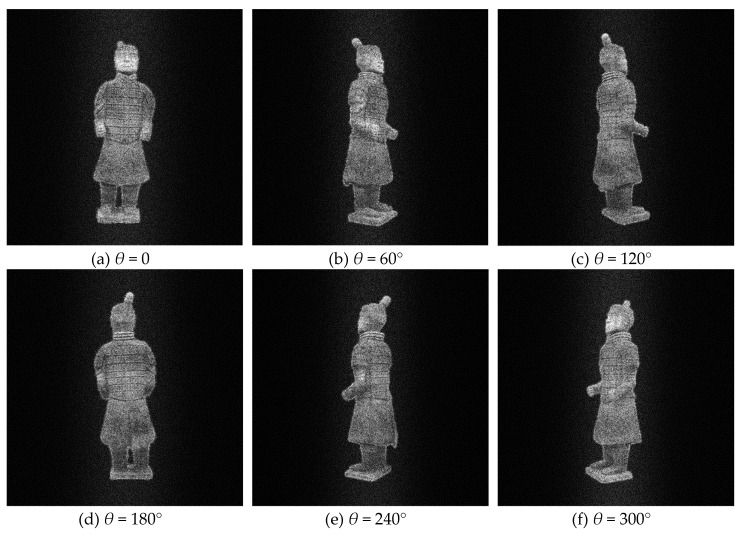
Digital reconstructed images at different viewing angles with kinoform sequence scheme I.

**Figure 6:**
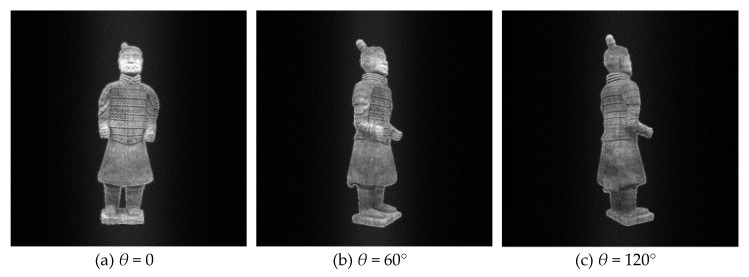

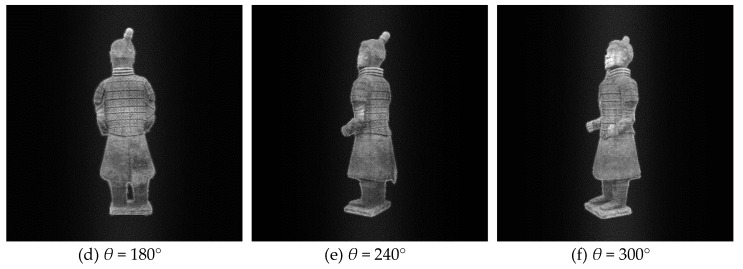
Digital reconstructed images at different viewing angles with kinoform sequence scheme II.

According to the previous theory, all reconstructed holographic images are superimposed on the final reconstructed plane in order to reduce the speckle noise caused by random phase. According to the experimental results, the more images superimposed at the same angle, the better the visual effect of the final synthesized image, the less noise contained in the image, and the better the texture details on the model can be seen. However, the more images are superimposed, the more computing time and tasks are needed in the computing process, and more storage space is needed. In practical application, the number of overlapped images can be adjusted according to the actual situation, because the human eye’s observation ability is limited. For this experiment, *T* is set as 20. Table 1 shows two redisplaying schemes. The first one is that, for each viewing angle, only one image is redisplayed from single hologram. The second one is a hologram sequence for each viewing angle. That is to say, *T* images for the same viewing angle with different random noises, and then these random noises are supressed by averaging effect. The original images from different perspectives are shown in Figure 4, the images redisplayd by the first scheme are shown in Figure 5, and Figure 6 shows the second scheme of reproducing the images. It is obvious that the noise in Figure 5 is more obvious than that in Figure 6. The reconstructed image in Figure 6 is visually clearer. 

**Figure 7:**
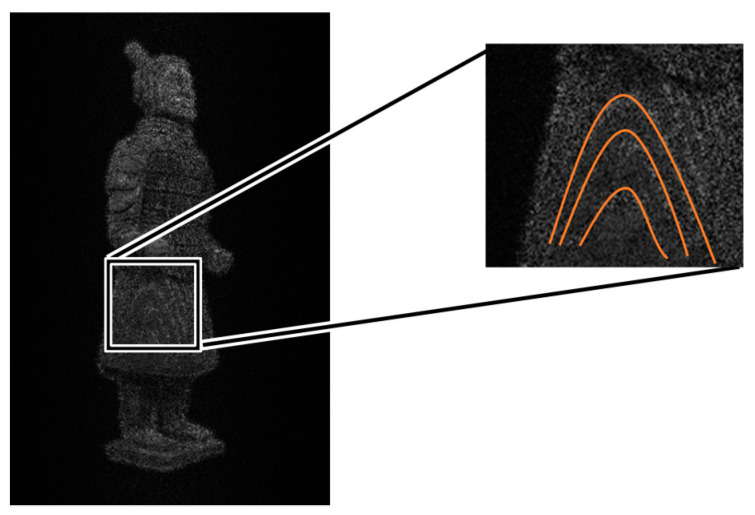
Layering Terra-Cotta Warrior model, thickness of layering set at 5 mm, and the inappropriate fringes are redisplayed on the model.

**Figure 8:**
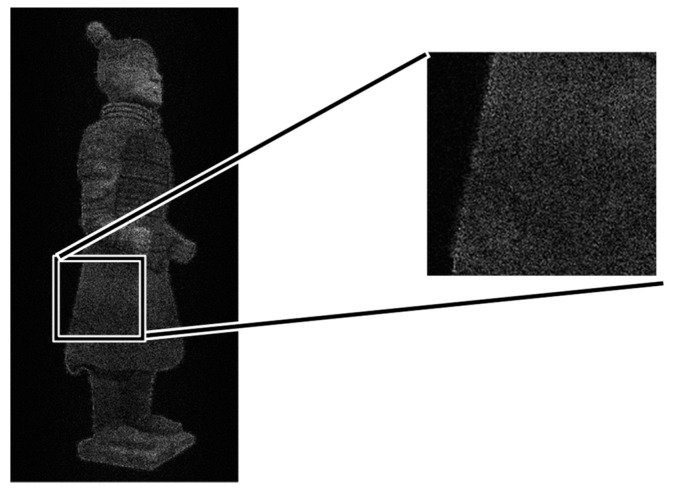
Layering Terra-Cotta Warrior model, thickness of layering set at 1 mm.

When we calculate the holograms by slicing the Terra-Cotta Warrior model with a thickness of 5 mm. Figure 7 shows the reconstructed image. The impact of these noises on our Terra-Cotta Warrior model is very significant, as we can clearly see the striped noise on the model. The thickness of layers is too large. When we slice the model with a thickness of 1 mm, the image obtained is shown in Figure 8. We can see that the fringe effect is weakened. It means that the layer thickness is sensitive to the imaging quality.

### 3.2. Electro-Optical Reconstruction Experiment

**Figure 9:**
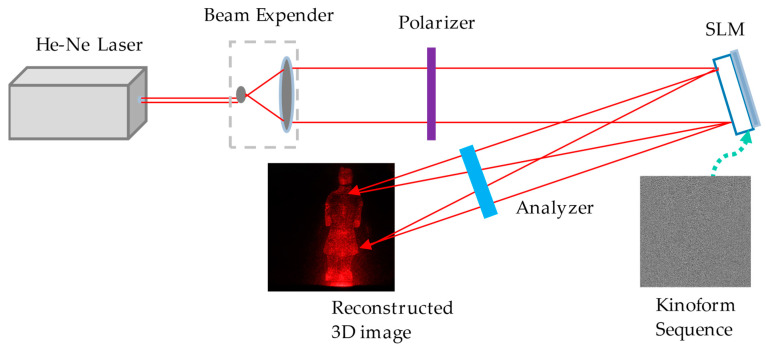
Experimental setup for dynamic 3D electro-optical reconstruction.

Figure 9 shows the experimental setup of dynamic 3D electro-optical reconstruction. He-Ne laser with power of 15 mW and wavelength 632.8 nm is used in the setup; the pixel number of the SLM is 1920 × 1080, and the refresh rate is 60 fps.

According to the display mode of our hologram, we also need to add a polarizer and an analyzer, which can adjust the working mode of SLM to phase mode. 

### 3.3. Image Quality Evaluation

We use a numerical speckle index (SI) [18] to evaluate the severity of image noise to evaluate the severity of image speckle noise. The formula for calculating SI is

(11) $$\mathrm{SI}=\frac{1}{MN}\overset{M}{\underset{m=1}{\sum }}\overset{N}{\underset{n=1}{\sum }}\frac{\sigma \left(m,n\right)}{\mu \left(m,n\right)}$$

**Figure 10:**
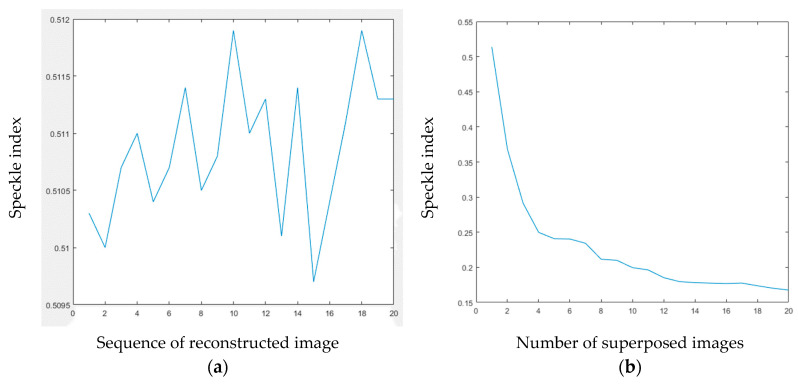
(**a**) Speckle index (SI) of reconstructed image from single hologram calculated by different random phases. (**b**) SI of superposed reconstructed images from different number of holograms.

In this formula, *M* × *N* is the size of image. σ(m,n) and μ(m,n) are used to calculate the standard deviation and average value of the 3 × 3 neighborhood around the image point *P*(*m*, *n*), respectively. Figure 10a illustrates the SIs of the reconstructed images from 20 holograms, which are holograms that are computed from the same image of the model, but with different random phases. Figure 10b shows the trend of SI of different number of superposed image reconstructed from the holograms. According to the formula, the smaller the SI index, the higher the quality of the representative image, and the smaller the noise. According to the changing trend of SI index in the graph, it can be seen that, with the increase of superimposed images, the SI index becomes smaller and smaller, and with the increase of image superimposition, the SI index decreases increasingly slowly.

### 3.4. Electro-Optical Reconstruction of Kinoforms

**Figure 11:**
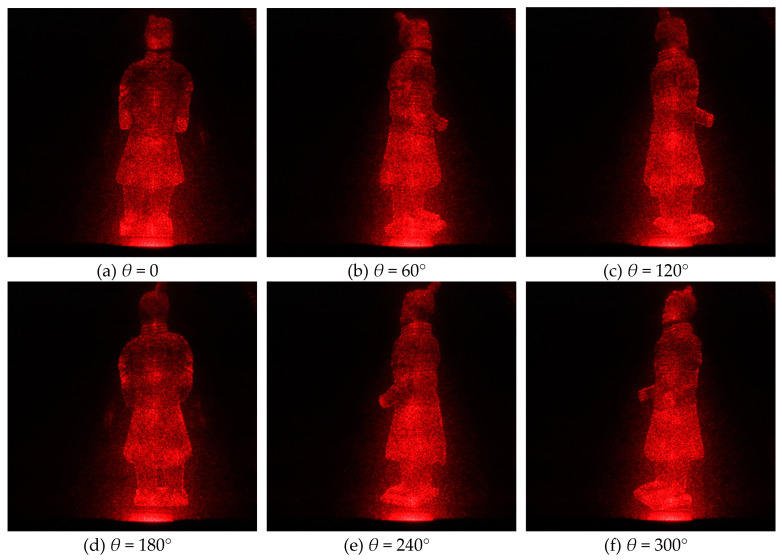
Electro-optical reconstructed images at different viewing angles with kinoform sequence scheme I.

**Figure 12:**
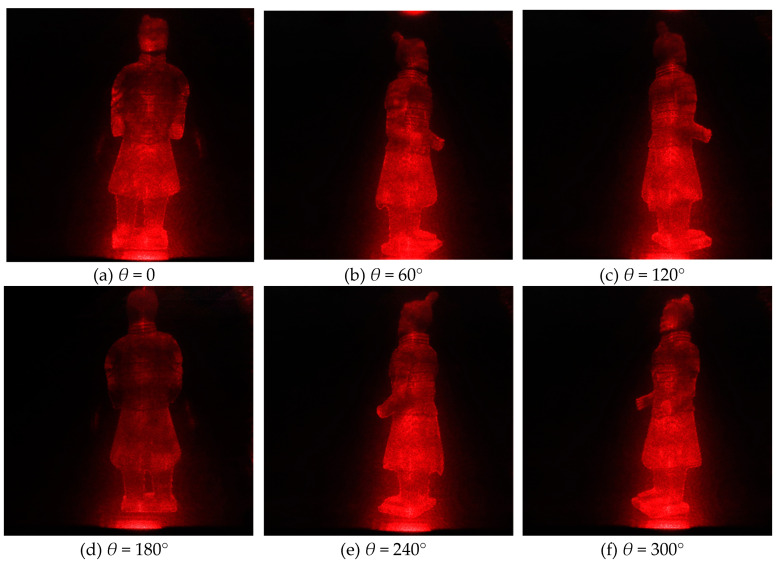
Electro-optical reconstructed images at different viewing angles with kinoform sequence scheme II.

We use this experimental device to redisplay the photoelectric image. Figure 11 is the observed 3D image when we use scheme I in Table 1. The hologram is loaded on SLM and then refreshed at 60 fps. The 3D image of the model is displayed. The reconstruction results that are shown in Figure 11 are from only one hologram at each angle, but the reconstruction results in Figure 12 are from 20 holograms at each angle. Obviously, in accordance with our simulation results, Scheme II can significantly suppress the speckle noise when compared with Scheme I.

For example, the SI of reconstructed image in viewpoint of 0° with scheme I is 0.2603; however, the SI of reconstructed image in viewpoint of 0° with scheme II is 0.1692. 

### 3.5. Relationship Between Computational Distance and Imaging Quality

According to the previous content, we successfully obtain the reconstructed image from the holograms of the model. In the experiment above, the distance that is used for calculating the image is 800 mm. When we calculate and simulate the reconstructed image, we evaluate the image quality of the same image at different imaging distances. In article [16], the relationship between speckle noise and the superimposed image is analyzed under two different imaging distances, but the trend of noise variation is not analyzed in detail. In this paper, we calculate many noise evaluation indices at different distances and analyze their changing trends. 

**Figure 13:**
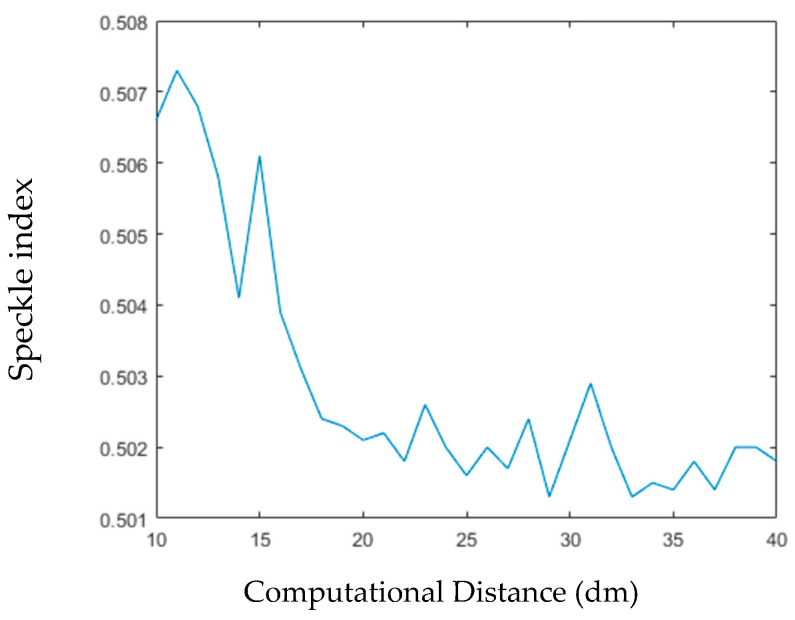
The Relationship between Computational Distance and Imaging Quality.

Figure 13 shows the relationship between imaging distance and image quality. Here, we still use SI to evaluate the image quality. As can be seen from the figure, the overall trend of image quality is increasing, because SI is decreasing. Some fluctuations can be seen in the statistical chart, which means that the curve is not smooth. The reason for these fluctuations is the random phase simulated by the image in the process of calculation. The noise trend that is generated by these random phases may be high or low, which leads to high or low image quality. 

Subsequently, we calculated the 10 phase holograms of single viewing angle at each computing distance. These holograms contain different phase distributions, which result in different random noises. We also calculated the SI of each reconstructed image, and the SIs of the 10 reconstructed images at the same distance are averaged.

**Figure 14:**
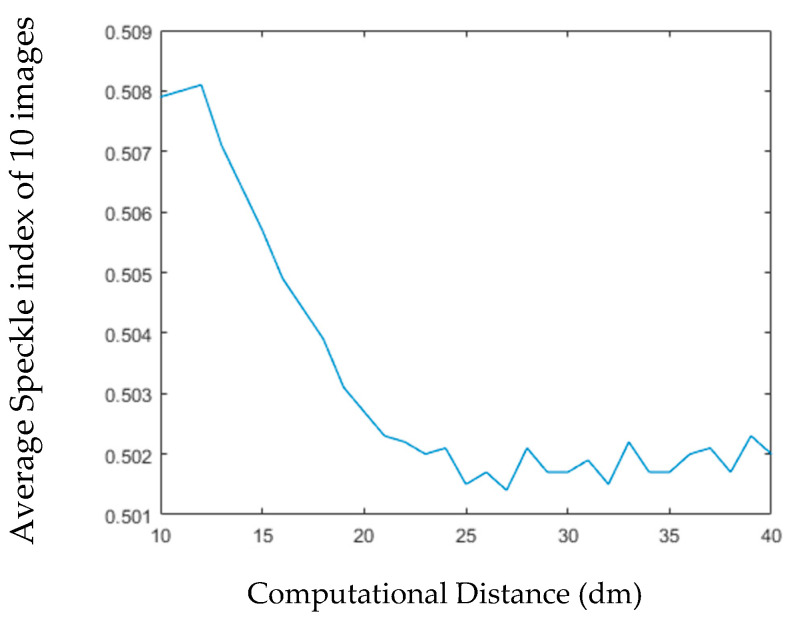
The Relationship between Computational Distance and Imaging Quality.

Figure 14 shows the relationship between the average SI of 10 images and the calculated distance. We can see that, the farther the calculation distance is, the lower the average SI is, and the trend of this curve is smoother than Figure 13.

**Figure 15:**
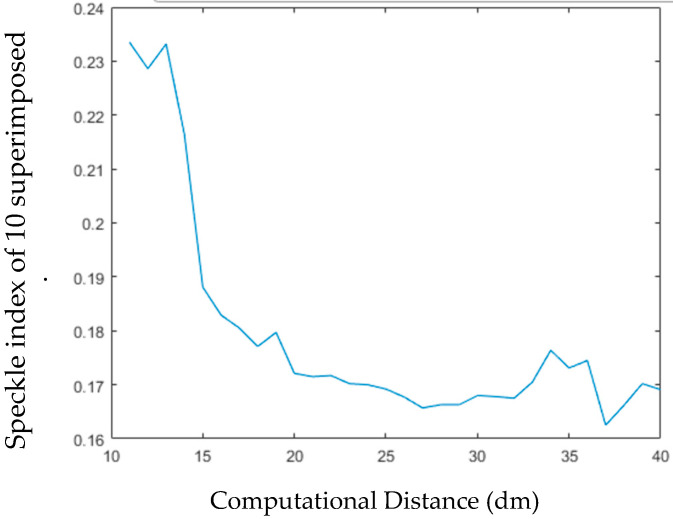
The Relationship between Computational Distance and Image Quality of 10 Superimposed Images.

Next, we calculate the SI of the superimposed images from 10 holograms for one viewing angle. In Figure 15, we calculate the SI of many different images at different computational distances. We can see from the graph that the quality of the image will be significantly improved, and the longer the distance is, the lower the SI is. The improvement of image quality may be the influence of depth of field is longer significant in the far computing distance than in the near computing distance. Accordingly, the image will be clearer.

## 4. Discussion

If we superimpose enough reconstructed images from the same angle, we can effectively reduce the speckle noise of image. However, the maximum refresh rate of SLM is 60 fps. That is to say, the longer the reconstructed image stays at the same angle, the slower the speed of three-dimensional display will be. Suppose that five holograms are used at one angle and a total of 60 viewpoints, it will take 5 seconds to display the whole holograms for the model, such as if 20 reconstructed images are superimposed from each angle, as in our Scheme II, 60-angle images need to be displayed, then it will take 20 seconds to see the whole reconstructed images of the model. In addition, as the number of superposed images increases, the computer needs increased time to calculate the hologram. We can reduce the number of superposed images to improve the display speed, but this will inevitably reduce the visual quality of the image. If we want to ensure the quality of the image and increase the display speed, we need to improve the refresh rate of the SLM. In addition, we discuss the relationship between imaging quality and calculating imaging distance as well as layer thickness. Ideally, the farther the distance is, the better the image quality is. 

## 5. Conclusions

In this paper, we introduce an advancement in FRT by calculating many noise evaluation indices at different distances and then analyze their changing trends while simultaneously responding in technical challenges of rough surfaces. When calculating the holograms of Terra-Cotta Warrior model by the slice-based method, the smaller the interval between layers, the smaller the speckle index and the more effective details can be shown. Otherwise, some bad stripes will be produced. At the same time, with the increase of computing distance, the image quality has been significantly improved, but the improvement of image quality is not obvious after a certain distance. An optoelectronic 3D image reconstruction system that is based on SLM is established. The phase holograms of a Terra-Cotta Warrior model with 60 viewpoints are calculated with an interval of 6°. In the process of image reconstruction, adding random phase on the object plane can significantly reduce the speckle noise by overlapping the reconstructed image.

## Figures and Tables

**Table 1:** Kinoform sequence schemes for dynamic three-dimensional (3D) holographic display.

Viewing Angle	0°	6°	…	354°
Scheme I$\left(H_{\theta ,t}\right)$	$H_{0,1}$	$H_{6,1}$	…	$H_{354,1}$
Scheme II$\left(H_{\theta ,t}\right)$	$H_{0,1},H_{0,2},H_{0,3},\ldots ,H_{0,T}$	$H_{6,1},H_{6,2},H_{6,3},\ldots ,H_{6,T}$	…	$H_{354,1},H_{354,2},H_{354,3},\ldots ,H_{354,T}$
